# Supplementary figures and images for: Late-rising CD4 T cells resolve mouse cytomegalovirus persistent replication in the salivary gland
Source: PLoS Pathog. 2024 Jan 18;20(1):e1011852. doi: 10.1371/journal.ppat.1011852 (PMC10796040; doi:10.1371/journal.ppat.1011852)

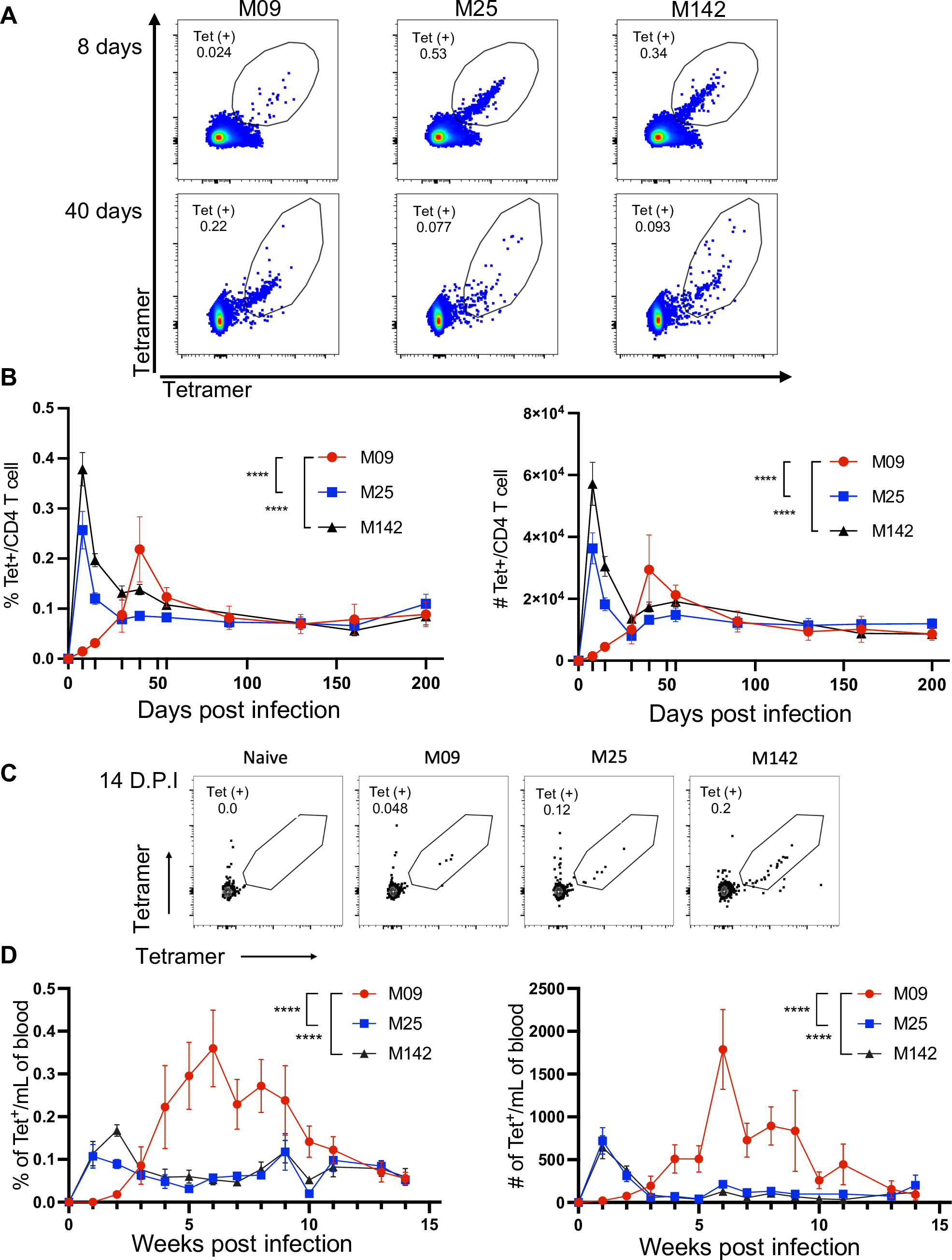

Supplement: S1 Fig — (A) Representative flow cytometry plot of M09, M25 and M142 double tetramer stained CD4 T cells in spleens at 8 and 40 D.P.I. (B) M09 (red dot), M25 (blue square) and M142 (black triangle) specific tetramer CD4 T cells in the spleen from day 0 to day 200 post infection, in percentage (left) and absolute number (right). (C) Representative flow cytometry plot of M09, M25 and M142 dual color-stained tetramer of CD4 T cells in the blood at 14 D.P.I. Blood from naïve mice were stained with the same tetramers, a representative staining of M09 tetramer is shown. (D) Specific tetramer CD4 T cells in the blood from day 0 to day 200 post infection, in percentage (left) and absolute number (right). The difference in kinetic was determined by Two-way Anova test. ****p < 0.0001. (TIF) [file ppat.1011852.s001.tif]

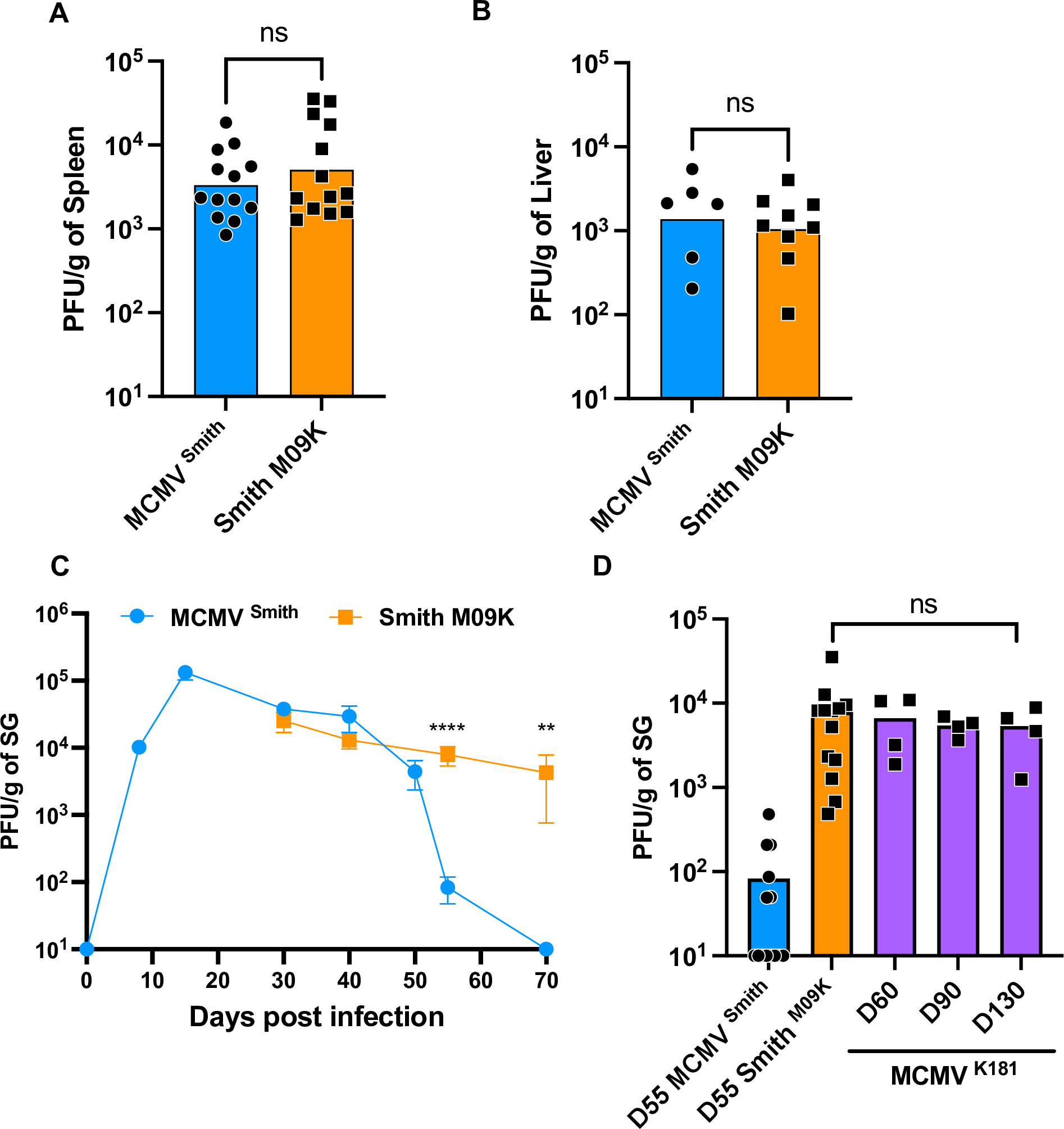

Supplement: S2 Fig — Replication of wild-type and Smith M09K in the spleen (A) and (B) liver at 4 days, and the SG (C) from day 30 to 70. (D) SG replication levels of MCMVK181 (60–130 dpi), and both MCMVSmith and Smith M09K at 55 dpi. Statistical significance was assessed by Mann Whitney test for A and B, and C was determined by Two-way Anova test. **p < 0.01, ****p < 0.0001. (TIF) [file ppat.1011852.s002.tif]

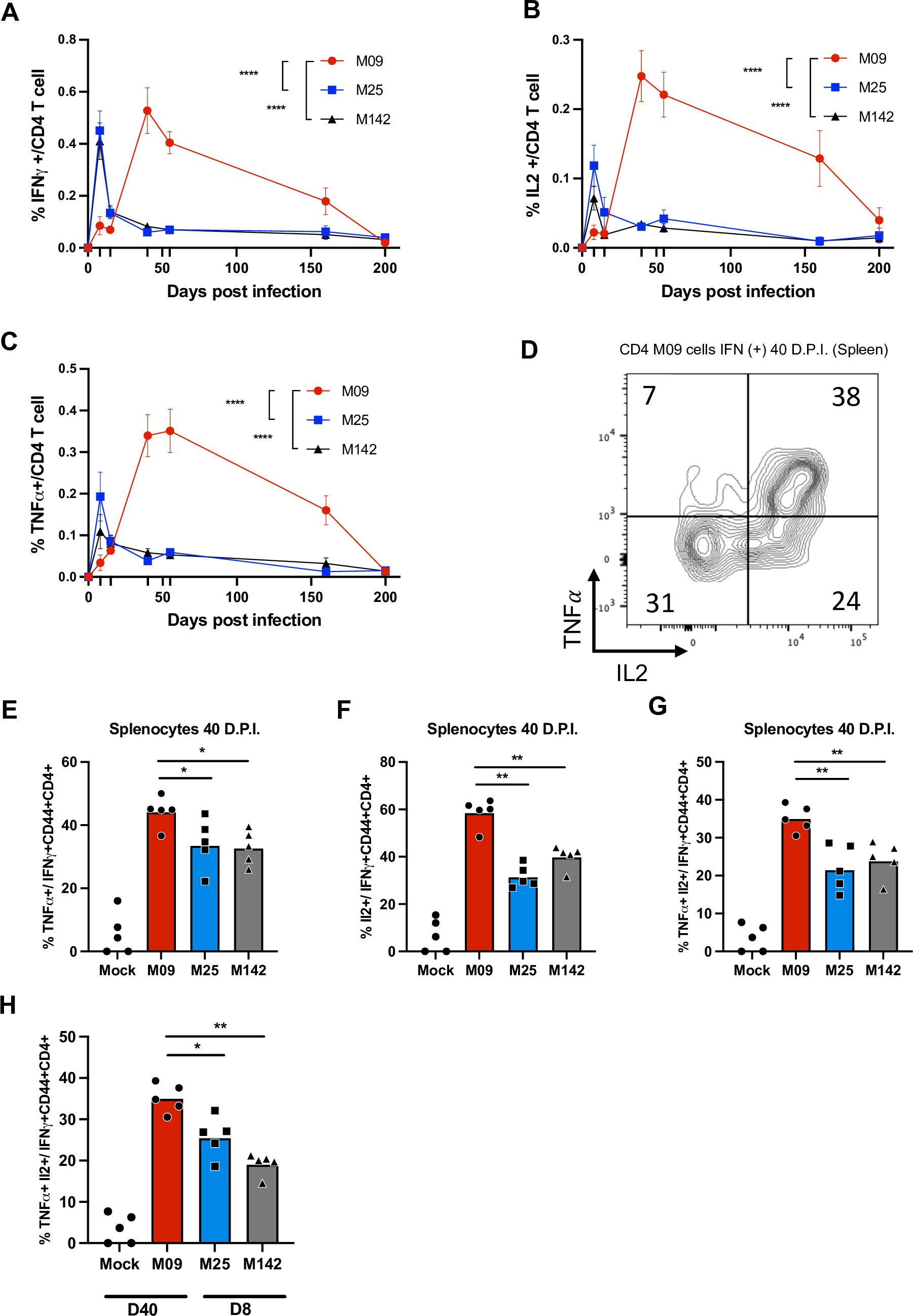

Supplement: S3 Fig — Kinetics of the percentage of CD4 splenocyte expressing I IFNγ (A), IL-2 (B) and TNFα (C) after peptide stimulation by M09 (red dot), M25 (blue square) and M142 (black triangle) peptide from day 8 to 200 of infection. Level of expression of TNFα (E), IL-2 (F) or both (G) cytokines among IFNγ memory CD4 T cells (CD44+) at 40 dpi from splenocyte MCMV peptide stimulation. (H) Percentage of triple positive memory (CD44+) CD4 T cells cytokine producer at day 40 (M09) and at day 8 (M25 and M142) post infection. (I) MFI of cytokine positive cells. Group comparisons were assessed by Mann Whitney test, *p< 0.1, **p< 0.01. (TIF) [file ppat.1011852.s003.tif]

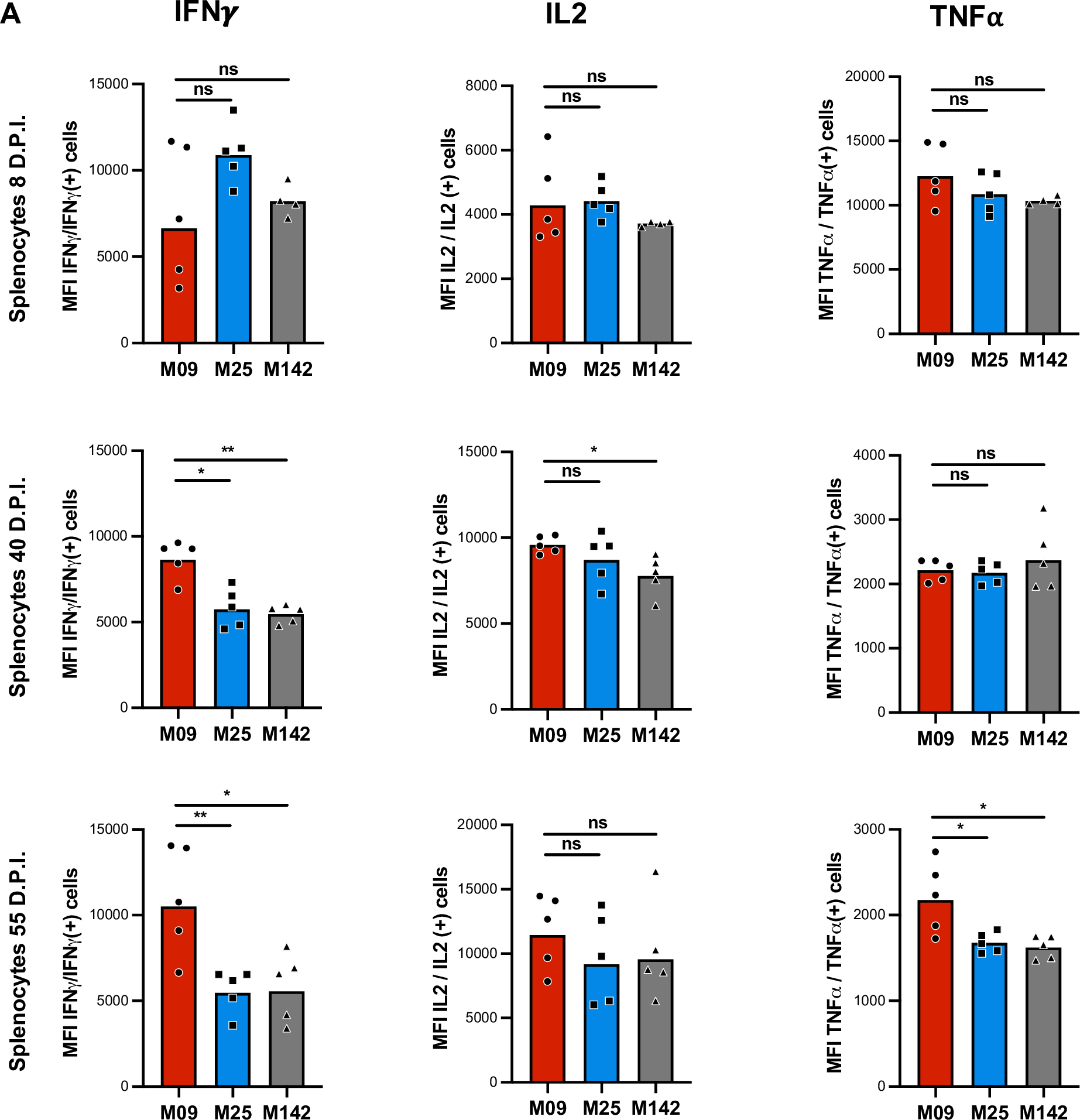

Supplement: S4 Fig — (A) MFI of cytokine positive cells for IFNγ, IL2 and TNFα at day 8, day 40 and day 55 post infection. Group comparisons were assessed by Mann Whitney test, *p< 0.1, **p< 0.01. (TIF) [file ppat.1011852.s004.tif]

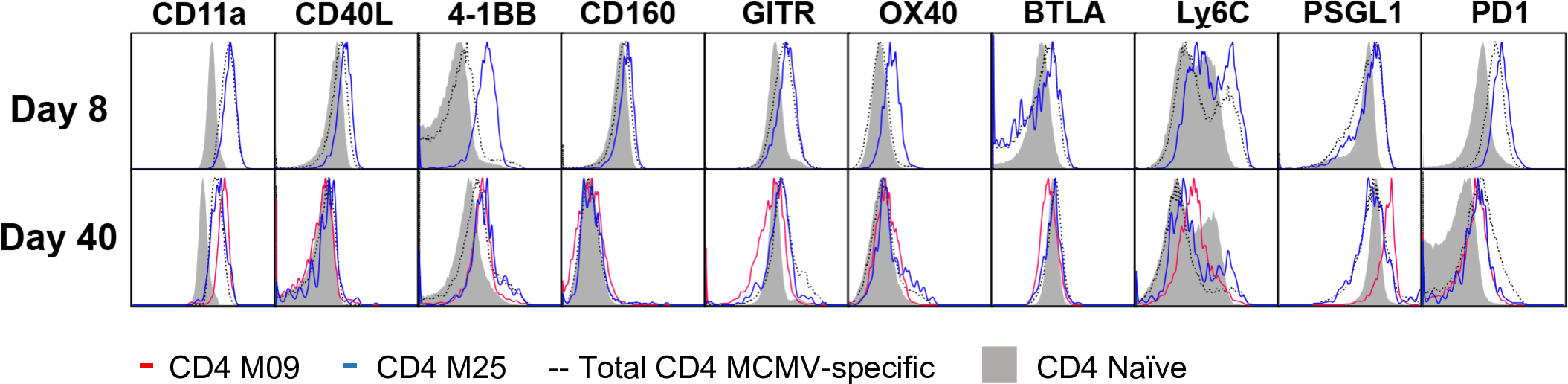

Supplement: S5 Fig — Cell surface markers expressed by conventional (M25, blue) and late-rising (M09, red) tetramer-binding CD4 T cells in spleen is shown at day 8 or 40 of infection. Total MCMV-specific (CD11ahiCD49d+, dashed histogram) and naïve (CD11aloCD49d-, gray filled histogram) CD4 T cells are also shown. (TIF) [file ppat.1011852.s005.tif]
