# Supplementary material for: Late-rising CD4 T cells resolve mouse cytomegalovirus persistent replication in the salivary gland
Source: PLoS Pathog. 2024 Jan 18;20(1):e1011852. doi: 10.1371/journal.ppat.1011852 (PMC10796040; doi:10.1371/journal.ppat.1011852)
Supplement: Attachment — Submitted filename: FINAL response to reviewers.pdf [file ppat.1011852.s006.pdf]

Summary

We feel it is a major plus that reviewer 1 stated that we had "…conducted elegant experiments to test their hypothesis, yielding interesting and compelling results", rev 2 believes that "…this study overall provides significant new insights into the MCMV model and immune control of CMV in general" and rev 3 thought that the study "…describes an interesting difference in the properties of immediate and late rising CD4 T cells".

We also feel positive that neither reviewer 1 or 3 had any major concerns with the study.

Reviewer 2 major issues:

1) *"The data in Figure 3B shows significant differences in the kinetics of the responses, but not whether the effector function at any timepoint is different. The conclusion that M09-specific cells are functionally superior would be strengthened by comparing the frequency tetramer+ cells at each timepoint, with those that produce cytokines. Or, alternatively/in addition, by the ratio of IFNg only producers, to producers of 2 or 3 cytokines."*

We agree with the reviewer that assessing the production of multiple cytokines in the conventional vs late-rising CD4T cells would add additional insight into the functional differences that M09 cells may exhibit. Consequently, we added Euler comparison charts for M09, M25 and M142 reactive cells for day 8 and day 40 post infection looking at the co-expression of IFNγ, TNF and IL-2 in these CD4 T cells (**new figures 3F, FigS3 E-H**), as well as the level of cytokine production per cell (**new Fig S3I**). Also, comparisons of the proportions of MCMV epitope-reactive CD4 T cells within the CD44+ population producing IFNγ have been added for 8 and 40 days post infection (**new figure Fig3E**). Finally, we added the MFI for cytokine expression as a new **figure S3I**. These new data are described in the results on lines 175-178.

2) *"The data in Figure 4 show differences in effector function at a transcriptional level, but at a single timepoint. This may be misleading since the M09 cells are peaking at d40, while M25-specific cells have long-since contracted. I wondered how the comparison of T cell function between M09-specific vs other T cells in Figure 3 would differ if compared at the peak of expansion."*

This is a legitimate point, and we have added some phenotypic/cytokine-production data in the new figure 3 (comment #1 above). We agree that having additional RNAseq data at various time points other than just day 40 would add additional insight, but respectively feel that this would be beyond the scope of the current work. We are currently initiating single-cell RNAseq experiments comparing M09 and M25 reactive CD4 T cells at various time points, as we also have the same questions as the reviewer. However, these experiments have just started, will take significant time to analyze once data is obtained and will be included in a follow up study.

3) *"Even if the M09-specific T cells are more functional on a per cell basis, I'm not sure how the vaccination data should be interpreted. There is currently no data to suggest that the vaccination induces M09-specific cells with altered effector function. Unless there is something special about the M09 peptide that promotes functionally distinct T cells, it would seem most likely that any improved function of M09-specific T cells is due to specifics of the MCMV infection context, which would be normalized by the vaccination platform. Nevertheless, vaccination with the M09 epitope reduced acute MCMV replication compared to other epitopes (Fig 5). Therefore, it seems possible (or maybe likely) that the efficacy of M09-specific T cells is related to the target, either as a superior epitope, or due to some unique feature*

of MCMV infected targets and how they express and present the M09 protein or epitope compared to other epitopes."

We agree with the reviewer, and believe that how the M09 protein is expressed during the course of acute/persistent infection, how the M09-derived epitope is processed and presented via MHC-II, the flavor of the APC that presents this epitope to CD4 T cells or another "infection related" issue underlies why M09 reactive cells arise late and display enhanced effector function in the context of infection and vaccination. Perhaps induction of M09 memory cells through vaccination allows for their earlier expansion when challenged with MCMV due to less stringent requirements than priming of naïve cells. At this time we don't have the clear answer, but are working on it for the future.

4) *"The frequencies and numbers of M25 and M142-specific T cells assessed by tetramer in figure 1 don't seem to parallel the data obtained by cytokine production in Figure 3. The cytokine data shows a much more prominent expansion and peak of the response at d8, followed by a sharp contraction compared to tetramer staining shown in 1C. It is hard to directly compare the data in these two figures. It would be helpful to show how tetramer+ staining compared with cytokine+ cells from the same mouse to know whether the tetramers or the cytokines were under or over-reporting on the cell numbers."*

The reviewer is 100% correct that the relative frequencies/numbers of late-rising CD4 T cells over a 200-day time course is more dramatic when assessing cytokine production following *ex vivo* epitope restimulation than when measuring the absolute # of tetramer+ cells. Our technique to analyze tetramer-binding cells (using tetramers labeled with two separate fluorophores and counting double+ cells) is the preferred method in the field, and is currently used by the Jenkins' lab who pioneered this technique. The greatest difference between these two methods is exhibited at the time of M09 peak expansion, when these cells are more "effector like" and M25/M142 are more "memory like", harkening back to point 2 raised by this reviewer. It is known that *ex vivo* restimulation of effector cells with peptide epitopes results in much faster cytokine production than that of memory cells (Jenkins MK et al., PMID: 11244029). which could partly explain these differences. We do restimulate for the standard time that most all in the field use (6 hours), and restim for 8 hours shows no difference in our hands. Consequently, we believe that both of our assays reliably identify M09, M25 and M142 reactive CD4 T cells, with these caveats that are issues for the entire field.

<u>Minor Issues:</u>

Reviewer #1: 1. Figure 2D – although the data are nice and go hand in hand with other results, one wonders whether mutations in K181 M09 would diminished tetramer binding and thus mask real M09-specific CD4 T cell response in mice infected with MCMV K181 and Smith M09K? If possible, authors should make a tetramer based on K181 M09 sequence and repeat staining. The result would not diminished their conclusions and the functionality of M09-specific CD4 T cells, however, it would provide relevant information.

This is a good point, and we also thought about this possibility. When we restimulated CD4 T cells from K181 infected mice with the analogous Smith $M09^{133-147}$ peptide epitope, we see statistically insignificant cytokine production compared to that in uninfected mice, correlating with our tetramer binding data. In addition, we generated a 15mer peptide pool (overlapping by 10mer) spanning the entire Smith and K181 M09 sequences, and saw no M09-derived epitopes targeted by CD4 T cells in K181 infected mice and only the 133-147 epitope for Smith. Consequently, we feel it is unlikely that other immunodominant epitopes derived from M09 exist in B6 mice.

2. Do M09Smith and M09K181-specific CD4 T cells differ in their functionality at early (day 8) and late

(day >40) time points? The functionality of CD4 T cells specific for both Smith and K181 M09 should be explored and tested for IFNγ, TNFα and IL-2 production.

As per question 1 above, there is no apparent M09-specific CD4 T cell response in K181 infected mice. In mice infected with the K181 M09S 'swap mutant', we see a robust CD4 T cell response directed against the Smith-derived M09 epitope (**figure 2D**).

3. Line 151 and Fig S2A and B – graph shows viral titer in spleen and liver from mice infected with MCMV Smith and Smith M09K only. Authors should also include viral titers of MCMV K181 and K181 M09S (as they mentioned in the text).

We changed the text to indicate this experiment was not performed in parallel groups of mice. Historically, we have always seen similar titers of wild-type Smith and K181 in the liver and spleen of acutely infected mice using either fibroblast-derived or SG-derived stocks of virus. Yes, K181 has been said to be more virulent, but over the years we haven't seen differences in acute-phase titers or LD50 as compared to Smith using BAC-derived viruses. The increased virulence manifests as longer persistent replication in our hands.

4. Figure S2C and D – the kinetics of MCMV K181 and K181 M09S might be shown also.

In Figure S2D we do show that replication levels of K181 are still maximal at day 130, more than 75 days beyond when Smith is controlled in most mice. Also, historically, we see similar levels of peak replication for K181 and Smith at day 15. We do not have a figure showing a similar time course for K181 as we do for Smith in Figure S2C, as this would take >4 months to complete. We would argue that given the data already shown in the manuscript it would not add significantly to the overall conclusions of the study, and respectively ask this would not be required for publication.

5. Figure 2D – include mock values

We have added a dashed line to this figure representing the background level of tetramer binding in uninfected B6 mice.

6. Although the data are nicely presented, the reader would benefit if more information on the graphs are shown; such as a type of tested organ (for example 1B, 1C, 1D, Figure 3, etc) or a legend (4A).

We have added this to the figures.

7. Line 146 – authors should clarify this better (I guess authors mean epitopes M25, M142 and M09).

We have clarified this better in lines 145-148, and are sorry for the initial confusion.

8. Figure 1B – one might find the layout of the graphs confusing.

The figure, legend and results section have been modified to reflect these changes.

9. Figure 1D – population percentage font size in dot plots should be bigger.

Done

10. Line 138 – the dot should be removed.

Done

Reviewer #2: *1) It appears that the data showing the Smith and K181 titers in the SG in Figure 2A and 2C are identical. If so, and the authors feel that it is essential to present the data this way, they should indicate this duplication in the legend. However, it appears to me that the data as presented in Fig 2C is sufficient to draw the conclusions as written in the text and 2A could be deleted unless it is from a distinct experiment.*

We felt that since the M09 epitope swap mutants were included in Fig 2C, the inclusion of data in Fig 2A again would be helpful for the reader. We have clarified this in the figure legend as well (line 629).

2) Please add information about how viral stocks were generated and maintained (i.e. tissue culture- or SG-derived virus?).

This information has been added to the M&M, we cited our past publication where we provided a very detailed description of our methods used to generate BAC-derived viral stocks (lines 314-315).

3) Fig 3A – The authors discuss in the text (lines 170-171) the frequencies of IL-2 producing T cells as a proportion of M09 or M25-specific T cells, but I believe they are referring to the frequencies of IL-2 or IFNγ producers among all CD4s, which is a different conclusion.

This was clarified in the figure legend

Reviewer #3:

1) Fig 1D is not referenced in the results text and the experiment/results are not detailed in the results.

Added Line 137.

2) Fig 2A shows MCMV persistence out to day 55 in the SG, have mice infected with K181 been studied longer than this, does the virus remain persistent or is it eventually controlled at much later times?

data up to day 130 is shown on Figure S2D

3) Could the authors comment on the apparent frequency disconnect between the tetramer data in Fig 1 and the frequency of M09 peptide cytokine producing cells. Fig 1C shows that approx. 10% of the CD4 cells in the SG are M09 positive while specific cytokine production was seen in only 0.2-0.5% Fig 3 B, C,D?

We are sorry for the confusion, figure 1C is data derived from the salivary gland, while 3B-D are from the spleen. We relabeled the figures and altered the legends to make this clearer.

4) Was a marker of activation also used in these experiments and if so are a large proportion of peptide activated CD4 T cells that are not making any of the cytokines measured?

SFig4 shows several different markers considered to be indicative of early/mid/memory-phase T cell antigen exposure (4-1BB, OX40, PD-1 etc). Additional canonical markers of T cell activation/memory are shown in Figure 4A. With regards to cytokine production, M25 cells are effectors at ~day 8 while M09 cells peak as effectors at ~day 40. This point was also raised by reviewer #2 in the ‘major issues’, please see our response above.

5) Have the M09 specific T cells been assessed for cytotoxic activity?

This is a very relevant question. To this point, we haven't assessed the cytotoxic potential of M09, or how it compares to M25/M142. We have established an *in vivo* cytotoxicity assay that was published in the past for the MCMV infection model (PMC4702662), and we plan to use it to address this question, although direct comparisons with conventional CD4 T may be difficult given the difference in expansion and effector kinetics. Additionally, we have initiated single-cell RNAseq experiments, and our first result does show cytolysis-associated genes expressed by splenic M09 cells at day 50 of infection (e.g. GzmK, TRAIL, CX3CR1, etc). However, we feel including any scRNAseq is premature and beyond the scope of this first paper.

6) The results show in Fig 4 and described in the text would benefit from some revision to help with understanding and clarity.

a. *Fig 4A I think the CD44/69/43 and 27 expression levels have been determined on M25 and M09 tetramer positive CD4 T cells but this is implied from the SFig3 figure legend, but is not stated in the Fig 4A figure legend, this could also be stated in the results eg line 180 M25 "tetramer specific" cells and line 186 M25 and M09 "tetramer specific" cells.*

   Clarified on lines 184 and 660

b. *The flow data in this figure using blur/red grey dashed and solid lines is very difficult to see.*

   The contrast has been increased to improve histogram resolution, and the labeling of the figure has been updated to make it simpler to interpret.

c. *Fig 4D/E please confirm this was tetramer staining of GFP positive (this IL-10 secreating) CD4 cells in SG.*

   Yes, this is CD43 vs IL-10/GFP+ expression in the M09 tetramer+ cells quantified in Fig 4D/E. We clarified this in the text lines, 311 and 365.

7) Fig 4F Y axis, should I think, be logs 10^3 to 10^5 PFU.

This has been changed

8) Fig 5A The "conv" vaccine preparation is a mixture of M25+M139+M142, M09 is added where the vaccines are compared. Results for M138 single vaccination are not show? In addition, are similar levels of the individual T cell responses induced when the combination of conv or conv+M09 are used as the vaccine (measuring individual responses by tetramer but administering the combined antigens) as this is the experimental vaccine condition being investigated?

Unfortunately, we did not determine the numbers of the M138 CD8T cells induced by vaccination as compared to CD4 T. We have very preliminary observations that the absolute numbers of splenic M09 and M25 cells is similar whether injected individually or together in the absence of an IFA boost and prior to infection, as well as data suggesting precursors in naïve mice are also similar (data not shown). Whether this remains true after infection hasn't been determined. This is now discussed in more detail on (lines 267-268).

9) Fig 5D While there is statistical significance when +/- anti IL-10R conditions are compared, for the key comparison at the 8 and 15 day time points, statistics are not shown for this, so it is unclear if it does test as significant please clarify? However, the point is well made that 3 of 4 and 2 of 4 animals in the CONV+M09 group completely controlled virus.

Statistics are now shown in figure 5D

10) The title omits that this is murine cytomegalovirus and that the persistence is in the SG?

We modified the title to reflect this, although we would prefer to say ‘persistence’ rather than ‘persistence in the SG’. Perhaps the reviewer would accept this?

While the discussion does mention the importance of CD4 cells in human and rhesus cytomegalovirus and suggests that vaccine strategies should consider CD4 T cell generation the implication from this MCMV work is that the peptide in M09 is somewhat unique in generating these late-rising T cells that control persistent replication and that this phenomenon might be mouse strain specific as well as wildtype MCMV isolates having mutations that do not lead to the generation of these T cells. Could the authors expand on their comments to give an idea of how unique the phenomenon might be to the B6 mouse strain? It is also somewhat difficult to imagine how the studies in HCMV CD4 T cells might be performed and maybe the authors could comment on this as well in their discussion.

We have added some thoughts with regards to this important question in the discussion lines 285-290.
